# Perceptions of Responsible Cat Ownership Behaviors among a Convenience Sample of Australians

**DOI:** 10.3390/ani9090703

**Published:** 2019-09-19

**Authors:** Alicia Elliott, Tiffani J. Howell, Emily M. McLeod, Pauleen C. Bennett

**Affiliations:** 1 Anthrozoology Research Group, School of Psychology and Public Health, La Trobe University, Bendigo, VIC 3552, Australia; aliciaelliott@outlook.com.au (A.E.); T.Howell@latrobe.edu.au (T.J.H.); Pauleen.Bennett@latrobe.edu.au (P.C.B.); 2 Department of Wildlife Conservation and Science, Zoos Victoria, Elliott Ave, Parkville, VIC 3052, Australia

**Keywords:** cat containment, wildlife protection, domestic cat management, desexing, *Felis catus*

## Abstract

**Simple Summary:**

Cat owners are responsible for keeping their pet cats safe, but some behaviors that keep cats and other animals safe, such as keeping the cat contained, are not supported by all owners. Non-owner attitudes towards responsible cat ownership behaviors are also important because cat owner behavior may be influenced by friends and family who do not own cats. Therefore, we asked owners and non-owners, recruited via social media sites associated with companion animal or wildlife issues, to describe their support for cat containment, microchipping, and neutering. Non-owners were more likely to report that cats should always be contained, but there was no difference in support for keeping cats contained at night. Owners were more likely to agree that cats should be neutered/spayed. In-principle support among owners was often higher than actual compliance with relevant behaviors, particularly for keeping cats indoors at night. This information may be useful for helping design campaigns to increase responsible cat ownership behaviors.

**Abstract:**

Responsible cat ownership is important for keeping pet cats and wildlife safe. Much research investigating levels of compliance with and attitudes towards responsible cat ownership practices has focused on cat owners. Non-owner attitudes are relevant because their opinions may encourage cat-owning friends and family to engage (or not) in a cat management practice. The aim of this study was to determine levels of compliance with responsible cat ownership practices among cat owners, as well as attitudes towards those behaviors by owners and non-owners alike. An online survey was completed by 6808 people living in Australia who were recruited via companion animal or wildlife interest groups on social media. Frequency data were used to measure owner compliance with responsible cat ownership behaviors and *t*-tests were used to determine whether owners and non-owners differed in their attitudes towards these behaviors. Owner compliance with responsible practices ranged from 46.5% (complete cat containment all day and night) to 76.9% (cat is de-sexed). Owner attitudes towards these practices were generally more positive than the reported levels of management practices implemented for their own cat. For example, 47.3% of owners agreed or strongly agreed that cats should always be contained and 88.6% agreed that cats should be contained at night. Non-owners were more likely than owners to agree that cats should be contained during the day, but there was no difference for containment at night. Owners were more likely to report that cats should be de-sexed. These results can be used to inform campaigns aimed at increasing compliance with responsible cat ownership behaviors.

## 1. Introduction

Responsible pet ownership requires that owners manage their pets to ensure community health and safety [1]. Cat owners must ensure good welfare for their cat while also helping to protect local wildlife populations from risk of predation. The Department of Environment, Land, Water and Planning in the Australian state of Victoria identifies cats as a major threat to wildlife populations [2]. Studies have suggested that pet, stray and feral cats kill millions of native vertebrates in Australia every year [3,4]. A comprehensive review of 93 studies on the predation of birds by feral and roaming pet cats in Australia estimated that cats kill 377 million birds every year [5]. Pet cats that roam outdoors were responsible for 16% of the predation of birds, which equated to 60.6 million birds predated upon by pet cats every year [5]. Cats that are not appropriately contained by their owners are also vulnerable to attack from other animals and traffic accidents [6,7]. They are perceived as a nuisance by owners and non-owners alike [8] and they can cause harm to the community through the spread of zoonotic diseases and parasites [6,7].

The Royal Society for the Prevention of Cruelty to Animals (RSPCA) in Australia identified three overlapping problems that may arise when domestic cats are not responsibly managed [9]. These are (i) the impacts on other animals, in particular native wildlife, (ii) the social impacts, such as public health issues or cats becoming nuisances, and (iii) the welfare impacts on the cats themselves [9]. Of the 21 recommendations that arose from this report to address these problems, six focused on increasing uptake of pet cat containment, de-sexing, and microchipping [9]. Microchipping can help increase reclaim rates of lost cats and help pounds, veterinarians, and other welfare organizations make informed decisions about lost cats in their care [9]. Increasing rates of de-sexing may help to reduce the stray cat population and reduce pound intake and euthanasia rates in Australia [9]. Reducing the cat population, increasing the number of cats reunited with their owners, and increasing the number of cats kept fully contained may improve the overall welfare of domestic cats and reduce the impact on Australian wildlife.

The RSPCA recognizes that any behavior change program needs to be based on an understanding of existing community attitudes and beliefs [9]. In one study in the state of Western Australia, 70% of participants showed support for de-sexing of cats not owned by licensed breeders [10]. This was irrespective of the participants’ area of residence (rural or urban), gender, or cat ownership status. This high level of support appears to translate into behavior when the cats are over the age of two years, with 93%–97% of cats assessed in a Western Australia sample found to be de-sexed [11]. However, there is less uptake of the behavior with younger cats, with only 28%–49% of cats aged less than two years old found to be de-sexed [11]. In two other studies exploring attitudes towards cat containment, one found that only 48% of cat owners supported cat containment, even though this is one of the most important ways to keep cats, and wildlife, safe [12]. A later study showed support for containment only at night (60%) or all the time (30%) among owners, while non-owners reported the reverse, with 61.5% supporting containment all of the time and just 28.5% supporting containment only at night [7]. In another study, attitudes which focused on owner stewardship and the benefits to pet cats of being brought inside were moderately correlated with intention to keep a cat indoors [13]. If the cat owner perceived that their household and veterinarian would believe that cats should be indoors, they were more likely to intend to keep their cat indoors [13].

Non-owner attitudes are potentially relevant in this context for two main reasons. First, since non-owners may encounter roaming cats in public spaces, and therefore may be forced to have interactions with them at least occasionally, they should be considered relevant stakeholders in any policy initiatives related to cat containment [8]. Second, their attitudes and opinions may influence the behaviors of cat-owning friends and family [14]. Indeed, there appear to be cultural considerations in attitudes towards cat containment; one study showed higher agreement with cat containment among Australian and Japanese owners and non-owners alike compared to respondents in the United Kingdom [15]. As such, understanding of non-owner attitudes could be valuable when developing campaigns designed to encourage cat containment. Among studies comparing owner and non-owner attitudes towards cat containment, support for containment is typically [15,16,17], but not always [18], higher for non-owners than for owners.

Most studies that compare cat owners and non-owners on attitudes towards cat containment do not measure actual cat containment among owners (see [7,15]). Furthermore, it is not always clear who cat owners rely on for information about cat management. In Australia, a country with unique native fauna that are at major risk of predation by cats [2], understanding the key influencers of attitudes and behaviors for cat owners is important to increase long-term compliance with cat containment. Therefore, the current study aimed to compare cat owners and non-owners in Australia on attitudes towards cat containment and trusted sources of information about cat care, as well as to measure owner compliance with cat containment. Additionally, following on from the RSPCA recommendations for domestic cat management, this study also aimed to examine attitudes towards and compliance with microchipping and de-sexing management behaviors.

## 2. Materials and Methods

### 2.1. Participants

A total of 6808 people in Australia completed an online survey. The majority (79.1%) were cat owners, and nearly all (91.6%) were female. The mean age was 41 years old (SD: 13 years; range: 18 to 93 years). The majority had some sort of post-secondary education, such as a university undergraduate (31.4%) or postgraduate (22.9%) degree or a trade/technical school certification (24.1%). Many were also in full-time (42.9%) or part-time (17.5%) paid work.

### 2.2. Materials

A questionnaire package was used, incorporating several demographic items and three existing scales, which were modified when needed (see Supplementary Material File S1). The results presented here form part of a larger survey. Results from components of the survey not presented here will be reported in future publications.

Participants reported their current cat ownership status. Then, they completed a survey section that was modified from the Dog Ownership Behaviors Questionnaire (DOBQ) [1]. This assesses individuals’ compliance with pet dog management behaviors and their attitudes towards dog ownership behaviors using belief statements. In this study, the DOBQ was modified to apply to cat owners’ performance of several cat ownership behaviors and participants’ attitudes towards cats and cat ownership behaviors.

If a participant owned a cat currently or had owned a cat in the last three months, they proceeded to answer questions based on the DOBQ, relating to their own cat. These questions were yes/no items about the performance of cat management behaviors (e.g., “Do you keep your cat indoors or in a cat run at all times”). Participants were also asked if they had ever lost a cat to a range of situations related to an outdoors lifestyle (e.g., car accident). These questions were located at the beginning of the survey to ensure that participants had not been primed by the content of the rest of the survey before they responded. All participants, including non-owners, were then asked to rate their level of agreement with belief statements about the performance of several management behaviors (e.g., “All adult pet cats should be kept indoors or in a cat run at all times”), as part of the modified DOBQ. We also asked for their level of agreement with items related to cats engaging in hunting and expressing natural behaviors. Then, we asked where participants would get information about cat keeping and care from (e.g., “Veterinarian”, “RSPCA”, “Friends”) and how likely they would be to act on advice from these sources, with a higher score indicating a greater likelihood of taking advice. Finally, demographic information was collected.

### 2.3. Procedure

Ethics approval was obtained from La Trobe University’s College of Science, Health and Engineering Human Ethics Committee (S17-063) prior to commencement of the study. Participants were recruited using the snowball method through Facebook, Zoos Victoria’s website, and a recruitment email sent to professional contacts of the research team. The organisations that shared the link to the survey on their social media pages included Anthrozoology Research Group, Zoos Victoria, RSPCA Victoria, RSPCA Australia, Threatened Species Commissioner, and Phillip Island Nature Park. Individuals who were under the age of 18 years or who did not reside in Australia were excluded from participation. The questionnaire was presented online using the survey platform Qualtrics (www.qualtrics.com) after each participant confirmed that they consented to participate in the survey, were at least 18 years of age, and resided in Australia. Cat owners who owned more than one cat were asked to choose the cat whose name started with the letter closest to the letter “A”. Completion of the questionnaire was completely anonymous and took approximately 20–30 min.

### 2.4. Analysis

Data were analysed using the Statistical Package for the Social Sciences (SPSS) Version 23 (IBM, Armonk, NY, USA). To determine how many cat owners permitted their cat to roam freely (i.e., not indoors, in a cat run, or on lead when outdoors), we calculated a variable combining an item detailing the times of day that the cat was most likely to be outdoors with an item asking where the cat spends most of its time when outdoors. Owners who indicated that the cat never free-roamed on both items were given a score of 1 (never) for the free roaming variable, owners who indicated that their cat may free-roam during the daytime were given a score of 2 (daytime only), and owners who reported that their cat may be free-roaming even at night were given a score of 3 (any time of day or night). To be conservative, we considered the items ‘restricted to property’ and ‘closely supervised’ as free-roaming since there is evidence that owners are unaware of where their cat goes [19]. However, if the owner stipulated in free-text that the cat was truly contained when outdoors (e.g., the cat could only access a balcony above the ground floor), then the cat was not considered to be free-roaming.

A series of independent samples *t*-tests were conducted to compare mean scores for the level of agreement with attitudes towards cat containment at night, containment at all times: microchipping, being de-sexed/neutered/spayed, and being allowed to express natural behaviours. Independent samples *t*-tests were also used to compare owners and non-owners on attitudes towards permitting cats to hunt, and sources of advice that would be relied upon regarding cat care and management. Given the large number of *t*-tests performed for some questions, Bonferroni corrections were applied when necessary.

## 3. Results

### 3.1. Owner-Reported Pet Cat Management Behaviours

Owners reported where their cat spent most of its time when outdoors and when during the day it would likely be outdoors. Nearly half (46.5%) of owners indicated that their cat was never free-roaming (i.e., always indoors, in a cat run or equivalent containment, or on lead). Another 29.3% indicated that their cat could be free-roaming during the day or at night, and nearly a quarter (24.3%) reported that their cat might be free-roaming, but only during the day.

We asked cat owners whether they had engaged in two other responsible cat ownership behaviours, finding that most owners indicated that their cat had been de-sexed (76.9%) and microchipped (72.2%). When asked about past cats, 66.3% reported that they had lost at least one cat to one of the listed incidents related to an outdoor lifestyle, including a third who reported they had lost a cat to a car accident (Table 1). It should be noted that it may be possible for some of these incidents to occur while a cat is contained or kept in a cat run, e.g., ingesting poisons, injury from wildlife, and falling from a height.

### 3.2. Attitudes Towards Responsible Cat Management Behaviours

Cat owners and non-owners were asked to indicate the extent to which they agreed with practicing several responsible cat management behaviors. For these items, a Bonferroni correction was applied, resulting in an alpha level of *p* < 0.01. As shown in Table 2, there was a significant difference between owners and non-owners on keeping cats indoors or in a cat run and de-sexing them, but no significant difference for microchipping them. Owners had stronger levels of agreement that cats should be permitted to express natural behaviours. A large majority (88.63%) of owners reported agreement (agree or strongly agree) that all pet cats should be contained at night time, while 47.29% agreed pet cats should be contained at all times, 95.66% agreed that pet cats should be microchipped and 93.9% agreed that pet cats should be de-sexed. For all items, the effect size was negligible or small, except for the item related to cats being permitted to express natural behaviors; the effect size for this item was medium.

### 3.3. Attitudes Towards Permitting Cats to Hunt

Cat owners and non-owners were asked a series of questions measuring their perceptions of cats hunting wildlife, as this could relate to beliefs that containment reduces cats’ ability to engage in natural behaviours. As shown in Table 3, owners were less likely than no n-owners to agree that hunting is a natural part of a cat’s behavior, although agreement for both groups was very high on this item. Owners were more likely to agree that hunting is important for a cat’s wellbeing, but agreement in both groups was very low. Cat owners and non-owners alike generally agreed that there is no need to permit a cat to hunt if it is well cared for. The effect size for all items was negligible or small.

### 3.4. Sources of Advice for Cat Care and Management

Participants who identified as current cat owners were asked to select from a list of sources which ones they currently go to or would go to for advice on cat care and management. The descriptive results are presented in Table 4. Veterinarians were the most accessed source, followed by a general google search and the RSPCA.

All participants were asked to indicate the likelihood that they would act on advice on cat care and management from a variety of sources. The descriptive statistics and *t*-test results are presented in Table 5. A Bonferroni-adjusted alpha level of *p* < 0.004 was applied. There were significant differences between owners and non-owners for most sources of advice, with the exceptions of a veterinarian, Google search, cat fancier association, and books. Non-owners obtained higher means for all sources except for social media and cat fancier association. For all the items, the effect size was negligible to small.

## 4. Discussion

The aim of the present study was to compare cat owner and non-owner attitudes towards cat containment and reliable sources of advice on cat care, as well as owner compliance with cat containment. A secondary aim was to measure compliance and attitudes towards de-sexing and microchipping. We obtained data from a large number of adults in Australia, but recruitment avenues employed likely mean that the results reflect attitudes among people who are concerned with companion animal or wildlife issues. This study, being undertaken in an Australian context, assumed that cat containment would be a desirable outcome for cats and wildlife, as Australia’s native fauna are under threat from cats [2], and Australians have high rates of support for cat containment compared to other parts of the world, such as the United Kingdom [15]. These assumptions may not hold in other regions.

A roaming lifestyle can be highly risky for cats, as evident from the two-thirds of cat owners in the study who reported having lost at least one cat to an incident related to roaming. The most commonly experienced risk was car accident, with a third of owners losing a cat this way, followed by almost a quarter of owners reporting having a cat who simply never came home. While some of the incidents listed (e.g., ingesting poisons or falling from a height) may have also occurred to a cat that was fully contained, cat owners who contain their cats have greater control over minimizing the occurrence of these risks than owners whose cats are free to roam unsupervised. Despite these risks, which had directly impacted two-thirds of the owners in this sample, less than half of cat owners agreed (47.29%) that pet cats should be kept indoors all the time, with about the same number of them reporting that they keep their current cat fully contained all the time. The majority (88.6%) of cat owners agreed that cats should be kept indoors at night, but only two-thirds of owners reported keeping their own cat indoors at night. This indicates an incongruity between a belief specific to the behavior and performance of the behavior itself.

Previous research in this area has suggested that cat owners perceive a low level of control over keeping their cat indoors, partly due to concerns about whether or not a cat kept purely indoors would be able to engage in naturalistic behaviors and thus be kept happy [13,20,21,22]. Indeed, cat owners were significantly more likely than non-owners to agree that cats should be able to express natural behaviors. While non-owners were more likely than owners to agree that hunting is one such natural behavior, owners had a stronger level of agreement that hunting was important for cats’ wellbeing. They were also less likely to agree that cats do not need to hunt provided they are well cared for. However, most differences in attitudes between owners and non-owners had a negligible to small effect size.

Concerns about being able to engage in natural behaviors while indoors may explain the disparity between attitudes and behavior reported in this study, as this has been reported in earlier research as a barrier to containment [22]. Within the last few years, mandatory cat confinement legislation has begun to be introduced in Victoria, the Australian Capital Territory and Western Australia, with cats required to be contained to their owners’ property [7,22]. However, owner compliance is difficult to regulate and while most cat owners agree with containment at night, not as many agree with permanent containment and the 24-h cat curfew laws being introduced [7,22].

Of all the owner-reported cat management behaviors, de-sexing was reported by the largest percentage of owners, followed closely by microchipping, with over three quarters of owners reporting that their cat was de-sexed (76.9%) and a similar percentage reporting their cat was microchipped (72.2%). Despite high rates of engagement with these behaviors, there was still an attitude-behavior gap, with nearly all participants agreeing that cats should be de-sexed (93.9%) and microchipped (95.7%). This gap may be a result of a few barriers to engaging in these behaviors, such as the costs associated with de-sexing or a vet visit to get a microchip, or owners waiting until the cat is older and/or had a litter before getting them de-sexed [4,9]. It is possible that the sample was biased, being self-selected and recruited using social media by organizations such as Zoos Victoria and the RSPCA. However, considering the large sample size in this study, it is likely that this level of de-sexing is indicative of de-sexing rates of pet cats in the general population. In fact, it is lower than previously reported rates of de-sexing in a representative sample taken from Victoria, Australia, in which 95% of respondents indicated having de-sexed their cat [23]. Perhaps the high rates reported here and in earlier research indicate success for vigorous educational campaigns to promote de-sexing [24] and substantially reduced registration rates for de-sexed cats in local councils [25].

To create behavior change, especially when the behavior is difficult to regulate as in this case, it can be useful to understand who the target audience considers to be a trusted source of advice, and who, therefore, could help facilitate the delivery of the behavior change message [26]. In this study, veterinarians were the most commonly accessed and trusted sources of advice on cat care and management, followed by the RSPCA. Friends, family, and a general Google search rated roughly in the middle, with social media and pet shops being the least likely to be relied upon. In addition to veterinarians and the RSPCA, there were a few other sources that appeared to be reasonably well trusted by cat owners, even if they did not report currently accessing them for cat care information. The majority were likely to accept advice from cat shelters or rescue centers other than the RSPCA, and approximately half the cat owners indicated they would be likely to act on advice from their local council (45.9%) or from Zoos Victoria, an Australian zoo-based conservation organization (54.2%).

In a study of veterinary clients in the United States, veterinarians were considered the most trustworthy source of information, as in the current study, followed by other pet owners with similar problems and then family/friends and the internet [27]. In a sample of visitors to Wellington Zoo, McDonald et al. [12] found that attitudes and normative beliefs were strong predictors of the participants’ intention to bring their pet cat inside at night. In particular, veterinarians were a key influence for participants who bring their cats inside [12]. This is supported by the results of the current study and highlights that both cat owners and non-owners view veterinarians as a trusted and credible source of cat care information. As such, campaigns aimed at increasing responsible cat management in Australia would likely benefit by working with veterinarians, the RSPCA, other pet shelters, and potentially local council/government bodies and zoos to deliver the message within the community.

While these were the sources most trusted for cat care information, these results do not distinguish between the type of cat care information. Depending on the context and message of the campaign, some of these sources may be seen as more credible sources of message delivery (e.g., veterinarians and the RSPCA over the council for a de-sexing campaign). Utilizing these trusted messengers and developing a persuasive communication strategy based on the best-practice principles of behavior change will help to increase the impact and effectiveness of campaigns to increase responsible cat management [28,29].

Our recruitment efforts were generally aided by conservation interest groups or companion animal interest groups, so it is likely that most of our participants were already knowledgeable about issues surrounding cat containment and protection of wildlife. Therefore, changing norms may be more important in this context than simply educating people about the topic. This makes understanding owners and non-owners preferred sources of information important. Veterinarians were the most trusted and accessed sources of information for both groups, but the RSPCA, other shelters and even local councils have the potential to act as messengers for campaigns promoting responsible cat ownership behaviors. Unlike some previous research [1,13], the attitudes and beliefs of both owners and non-owners were surveyed in the current study. This is important because any targeted campaigns seeking to change cat owner behaviors will be more likely to succeed if they increase perceived normative pressure to perform the cat management behaviors [1]. Indeed, cultural differences reported in one study suggest that societal norms play a large role in compliance with, and attitudes towards, cat containment [15]. Hence, both non-owners and cat owners alike can be involved in changing attitudes [1]. Knowing what the public currently believes about cats and cat ownership behaviors is vital to be able to properly address these attitudes through campaign materials. Understanding which sources of information are considered trustworthy can also help inform how these materials are disseminated.

### Future Directions

The large sample size of this study indicates considerable interest in the discussion of responsible keeping of pet cats by cat owners and non-owners alike. This is important as effective cat management requires a community response as well as an individual one. While a strength of this study was its large sample size, approximately 93% of the sample was female. This can make it more difficult to generalize the findings to the general population. However, the size of the sample, and the fact that approximately 200 males did complete the study makes it one of the largest studies of male attitudes towards cats conducted. Further analysis of gender differences was beyond the scope of this report but is planned for the future. Similarly, due to the scope of the current project, very few open-ended questions were included in the survey, and this is something that should be rectified in future studies to investigate reasons for compliance with the behaviors in greater depth.

The organizations that promoted this survey are likely to attract followers who are animal-friendly, but not necessarily cat-friendly (e.g., the Threatened Species Commissioner’s social media outlets are likely to be followed by many Australians who worry about the effect of cats on wildlife). Future research should aim to obtain a representative sample of Australians, capturing participants without strong feelings towards cats.

## 5. Conclusions

Support for responsible cat ownership behaviors, such as containment, de-sexing, and microchipping of pet cats, was high in this very large sample of Australian adults; however, there were significant differences between cat owners and non-owners on level of support for most cat management practices. Furthermore, rates of actual responsible cat ownership behaviors were lower than might be expected, since owner perceptions of these behaviors were generally very positive. Owners and non-owners also differed on beliefs about whether the ability to hunt is important for cats. However, owners and non-owners both rated veterinarians as the source of information about cat care that they would be most likely to accept. The results of this study can be used to inform campaigns about responsible cat ownership.

## Figures and Tables

**Table 1:** The percentage of cat owners in this sample who reported having lost a cat in the past to each of the situations listed below related to an outdoor lifestyle. Participants were able to select multiple responses.

	%	N
Car Accident	34.1	1833
Feline Immunodeficiency Virus (FIV)	5.1	275
Dog attack	7.4	396
Human attack	3.3	178
Skin cancer	5.7	307
Injury from wildlife (e.g., snakebite)	7.3	395
Ingesting poisons (e.g., rat bait)	3.6	191
Falling from a height (e.g., out of a window or tree, or off a ledge)	1.1	61
Unknown, my cat never came home	23.6	1272
Other	17.8	959
None of the above	33.8	1818

**Table 2:** Means (*M*), standard deviations (*SD*), percentage (%) of respondents who either agreed or strongly agreed with specific statements and *t*-test results comparing cat owners and non-owners on a series of responsible cat management variables. Cohen’s *d* (d) effect sizes are also presented. Items were measured on a 5-point Likert scale, with higher scores indicating a higher level of agreement with the item.

Agreement-All Pet Cats:	Owners			Non-Owners			*t*	*df*	*p*	*D*
	*M*	*SD*	*%*	*M*	*SD*	*%*				
Should be kept indoors or in a cat run at night time	4.50	0.88	88.6	4.57	0.81	86.8	−2.70	2306.2	0.007 *	0.08
Should be kept indoors or in a cat run at all times	3.35	1.35	47.3	3.66	1.37	56.9	−7.47	6250	<0.001 *	0.23
Should be microchipped	4.67	0.63	95.7	4.69	0.58	89.0	−0.97	2245.2	0.332	0.03
Should be de-sexed/neutered/spayed	4.64	0.68	93.9	4.56	0.77	81.0	3.35	1855.2	0.001 *	0.11
Should be allowed to express natural behaviours	3.99	0.77	78.7	3.52	1.07	53.6	14.64	1607.9	<0.001 *	0.50

* difference is significant at a Bonferroni-adjusted alpha level of *p* < 0.01.

**Table 3:** Means (*M*), standard deviation (*SD*), percentage (%) of respondents who either agreed or strongly agreed with specific statements, and *t*-test results comparing cat owners and non-owners on a series of items related to perceptions of hunting by cats. Cohen’s *d* (d) effect sizes are also presented. Items were measured on a 5-point Likert scale, with higher scores indicating a higher level of agreement with the item.

Agreement:	Owners			Non-Owners			*t*	*df*	*p*	*D*
	*M*	*SD*	*%*	*M*	*SD*	*%*				
Hunting is a natural part of a cat’s behaviour	4.17	0.77	88.4	4.33	0.75	93.2	−7.13	2347.3	<0.001 *	0.21
Hunting is important for a cat’s wellbeing	2.53	1.10	19.7	2.30	1.11	17.6	6.70	6371	<0.001 *	0.21
If a cat is well cared for, there is no need to allow it outside to hunt	3.95	1.06	70.6	4.01	1.07	73.1	−1.96	6370	0.050	0.06

* difference is significant at a Bonferroni-adjusted alpha level of *p* < 0.017.

**Table 4:** The percentage of cat owners in this sample who go to or would go to the various listed sources for cat care and management advice. Participants were able to select multiple sources.

Source:	%	N
Veterinarian	78.96	4251
General Google Search	51.37	2766
RSPCA	43.67	2351
Other cat shelter or rescue (e.g., Cat Protection Society, Lost Dogs Home)	34.27	1845
Friends	29.98	1614
Books	28.90	1556
Family	28.01	1508
Local Council	17.72	954
Social media	15.43	831
Cat Breeder	14.67	790
Pet Shop	13.89	748
Zoos Victoria	6.37	343
Cat Fancier Association	4.14	223

**Table 5:** Means (*M*), standard deviations (*SD*), and *t*-test results comparing cat owners and non-owners on likelihood of accepting advice on cat care and management from various sources. Cohen’s *d* (d) effect sizes are also presented. Items were measured on a 5-point Likert scale, with higher scores indicating a higher likelihood of relying on that source of advice. Items are listed in order of highest to lowest M for owners.

Likelihood of Acting on Advice From:	Owners		Non-owners		*t*	*df*	*p*	*D*
	*M*	*SD*	*M*	*SD*				
Veterinarian	4.65	0.59	4.66	0.98	−0.23	5700	0.817	0.02
RSPCA	4.00	0.89	4.17	0.87	−5.87	1941.5	<0.001 *	0.19
Cat shelter or rescue other than RSPCA (e.g., Cat Protection Society; Lost Dogs Home)	3.85	0.81	3.91	0.84	−2.26	5646	0.024	0.07
Books	3.67	0.78	3.68	0.82	−0.24	5643	0.810	0.01
Zoos Victoria	3.47	1.00	3.61	1.03	−4.45	5638	<0.001 *	0.14
Family	3.41	0.81	3.52	0.81	−4.15	5648	<0.001 *	0.14
General Google search	3.38	0.79	3.39	0.81	−0.35	5652	0.727	0.02
Friends	3.34	0.78	3.47	0.79	−5.11	5648	<0.001 *	0.17
Cat breeder	3.30	1.09	3.44	1.07	−3.89	5645	<0.001 *	0.13
Local council	3.28	0.91	3.64	0.87	−12.54	1967.2	<0.001 *	0.40
Cat fancier association	3.04	0.98	3.02	1.00	0.78	5626	0.435	0.02
Social media	2.84	0.88	2.72	0.89	3.88	1860.9	<0.001 *	0.14
Pet shop	2.71	1.07	2.84	1.05	−3.67	1914.7	<0.001 *	0.12

* Difference is significant at Bonferroni-adjusted alpha level of *p* < 0.004.

## Supplementary Materials

The following are available online at https://www.mdpi.com/2076-2615/9/9/703/s1, S1: Full survey.
