# Supplementary material for: Perceptions of Responsible Cat Ownership Behaviors among a Convenience Sample of Australians
Source: Animals (Basel). 2019 Sep 19;9(9):703. doi: 10.3390/ani9090703 (PMC6769723; doi:10.3390/ani9090703)
Supplement: Supplementary file 1 [file animals-09-00703-s001.pdf]

Living with Cats: Attitudes Towards Cat Ownership

Do you own, or have you owned, one or more cats in the last 3 months?

- ◯ Yes
- ◯ No

What is this cat's name? (If you have more than one cat, please write the cat whose name starts with the letter closest to the letter A.)

____

What sex is [cat’s name]?

- ◯ Male
- ◯ Female

How old is [cat’s name]?

- ◯ Months ____
- ◯ Years ____

How old was [cat’s name] when you acquired him/her?

- ◯ Days ____
- ◯ Months ____
- ◯ Years ____

What was the main reason you acquired [cat’s name]?

- ◯ Companionship
- ◯ The cat needed a home
- ◯ To keep other pets company
- ◯ For your children
- ◯ For pest/rodent control
- ◯ To keep other cats away from the home
- ◯ Other ____

Where did you obtain [cat’s name]?

- ◯ Pet shop
- ◯ Breeder
- ◯ Animal shelter
- ◯ Friend/Relative
- ◯ Advertisement in a newspaper or shop
- ◯ Found him/her
- ◯ Bred him/her myself
- ◯ As a gift
- ◯ Other ____

How much did [cat's name] cost? If you do not remember, please give your best estimate.

- ◯ $0
- ◯ $1-$100
- ◯ $101-$500
- ◯ $501-$900
- ◯ $901-$1300
- ◯ $1301-$1700
- ◯ $1701-$2100
- ◯ >$2101

Are you primarily responsible for the care of [cat's name]?

- ◯ Yes
- ◯ No
- ◯ Shared equally

Is [cat's name] a pure breed?

- ◯ Yes
- ◯ No

Before you got [cat's name], how long had you been planning to obtain a cat?

- ◯ No time at all (I acquired the cat on impulse)
- ◯ About a month
- ◯ About 2 or 3 months
- ◯ About 6 months
- ◯ About a year
- ◯ Longer than one year

In your lifetime, about how many cats have you lived with (including current cat/s)?

____

Do you currently own any pets other than [cat's name]?

○ Yes

○ No

Is [cat's name]...?

	Yes	No	Not Sure
Registered with your local council	○	○	○
Microchipped	○	○	○
Up to date on his/her vaccinations	○	○	○
Desexed/Neutered/Spayed	○	○	○

Does [cat's name] receive annual health checkups from a veterinarian?

○ Yes

○ No

○ Not Sure

On a typical day, for each of the following time periods, please indicate how often [cat’s name] is likely to be outdoors:

	Never	Some of the time	About half the time	Most of the time	Always
Midnight - 4am	○	○	○	○	○
4am - 8am	○	○	○	○	○
8am - Noon	○	○	○	○	○
Noon - 4pm	○	○	○	○	○
4pm - 8pm	○	○	○	○	○
8pm - Midnight	○	○	○	○	○

When [cat’s name] is outdoors where does s/he spend most of his/her time?

- ○ Not applicable - [cat’s name] is solely inside
- ○ Restricted to a cat run
- ○ Restricted to my property
- ○ Closely supervised
- ○ Free roaming
- ○ On a leash
- ○ Other ____

When [cat’s name] is outdoors, how far from home do you think [cat’s name] regularly travels?

- ○ 1m - 50m
- ○ 50m - 100m
- ○ 100m - 500m
- ○ 500m - 1km
- ○ 1km - 5km
- ○ 5km-10km
- ○ >10km
- ○ I don't know

To what extent do you agree or disagree with the following statements about allowing [cat’s name] outdoors?

	Strongly disagree	Disagree	Neither agree nor disagree	Agree	Strongly agree
I don't see any problem in allowing [cat’s name] free access to the outdoors	○	○	○	○	○
I am confident [cat’s name] doesn't catch any wildlife when allowed outside	○	○	○	○	○
I am aware of everything [cat’s name] catches	○	○	○	○	○

How often do you think [cat’s name] catches wildlife?

- ◯ At least once a day
- ◯ Once per week
- ◯ Once per month
- ◯ Once every few months
- ◯ Once a year
- ◯ Never
- ◯ I don't know

How concerned are you that if [cat’s name] was outside she/he may:

	Not at all concerned	Slightly concerned	Somewhat concerned	Moderately concerned	Extremely concerned
Kill introduced (non-native) species	○	○	○	○	○
Kill native wildlife species	○	○	○	○	○

If [cat’s name] is allowed outdoors, how concerned are you about the following risks?

	Not at all concerned	Slightly concerned	Somewhat concerned	Moderately concerned	Extremely concerned
Risk of injury from a car accident	○	○	○	○	○
Risk of injury from fights with other cats	○	○	○	○	○
Risk of injury from dog attack	○	○	○	○	○
Risk of injury from humans	○	○	○	○	○
Risk of upsetting neighbours due to [cat’s name] wandering	○	○	○	○	○
Risk of sunburn/cancer	○	○	○	○	○
Risk of injury from wildlife (e.g. snakebite)	○	○	○	○	○
Risk to wildlife	○	○	○	○	○
Risk of being stolen	○	○	○	○	○

To what extent do you agree or disagree with the following statements about keeping [cat’s name] indoors?

	Strongly disagree	Disagree	Neither agree nor disagree	Agree	Strongly agree
It is important to provide [cat’s name] with toys and other types of stimulation, to keep him/her happy indoors	○	○	○	○	○
I am confident that I can provide everything to ensure [cat’s name] is happy indoors or in a cat run at all times	○	○	○	○	○
I am confident I could contain [cat’s name] indoors or in a cat run at all times	○	○	○	○	○

Please indicate the extent to which the following considerations influence your decision to **not** keep [cat’s name] indoors all the time:

	Not at all influential	Slightly influential	Somewhat influential	Very influential	Extremely influential
Cleaning the litter tray	○	○	○	○	○
[cat’s name] would annoy me trying to get out	○	○	○	○	○
[cat’s name] would be bored if s/he stayed inside all the time	○	○	○	○	○
[cat’s name] may spray (inappropriately urinate indoors) if I didn't let him/her out	○	○	○	○	○
[cat’s name] wants to go outdoors	○	○	○	○	○
My personal preference	○	○	○	○	○
Cats belong outside	○	○	○	○	○
It's what I've always done	○	○	○	○	○
My friends/family think cats should be outside	○	○	○	○	○
Other	○	○	○	○	○

What would encourage you to keep [cat’s name] indoors at all times?

____

Have you ever lost a cat to: *(please tick all that apply)*

- ☐ Car Accident
- ☐ Feline Immunodeficiency Virus (FIV)
- ☐ Dog attack
- ☐ Human attack
- ☐ Skin cancer
- ☐ Injury from wildlife (e.g. snakebite)
- ☐ Ingesting poisons (e.g. rat bait)
- ☐ Falling from a height (e.g. out of a window or tree, or off a ledge)
- ☐ Unknown, my cat never came home
- ☐ Other ____
- ☐ None of the above

If [cat’s name] is allowed outdoors, which of the following factors could make you want to exclusively keep him/her indoors?

	Extremely unlikely	Unlikely	Neither likely nor unlikely	Likely	Extremely likely
Improving [cat’s name]'s mental health	◯	◯	◯	◯	◯
Improving [cat’s name]'s physical health	◯	◯	◯	◯	◯
Increasing [cat’s name]'s life span	◯	◯	◯	◯	◯
Helping native wildlife	◯	◯	◯	◯	◯
Reducing risk of harm to [cat’s name] from other humans or dogs	◯	◯	◯	◯	◯
Reducing risk of harm to [cat’s name] from wildlife	◯	◯	◯	◯	◯
Giving you more one-on-one time with [cat’s name]	◯	◯	◯	◯	◯
Improving the relationship/bond [cat’s name] has with you	◯	◯	◯	◯	◯

Which of the following, if any, do you provide to [cat's name] when she/he is indoors *(Please tick all that apply)*:

- ☐ Other cats in the home
- ☐ Animals other than cats in the home
- ☐ Scratching post
- ☐ Window to look out of
- ☐ Access to fresh air (e.g. through an open window)
- ☐ Access to sunlight
- ☐ Litter tray
- ☐ An elevated place to sit
- ☐ TV left on
- ☐ Unrestricted access to the outdoors
- ☐ Access to an enclosed outdoor space (e.g. cat run)
- ☐ Food treats
- ☐ Clean drinking water
- ☐ Play gym
- ☐ Toys and/or puzzles
- ☐ Other ____

What type of food does [cat's name] <u>usually</u> eat in an average day?

- ☐ Canned food
- ☐ Dry food
- ☐ Home prepared food
- ☐ Commercially prepared raw diet
- ☐ Meat and/or bones
- ☐ Other ____

How many meals is [cat's name] fed per day?

- ○ Less than once
- ○ One
- ○ Two
- ○ Three or more
- ○ Food is always available for my cat

What types of snacks (e.g. Greenies, Liver treats etc), and how many, if any, does [cat's name] usually receive in an average day?

____

Based on the pictures below, please indicate the picture which best represents [cat’s name]'s body shape.

○ 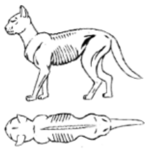

○ 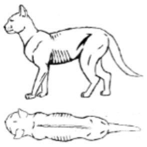

○ 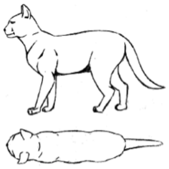

○ 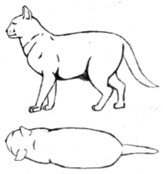

○ 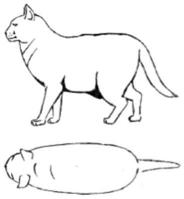

Please consider each of the following words and indicate to what degree it describes [cat’s name]'s personality:

	Describes [cat's name] NOT AT ALL	Describes [cat's name] ONLY SLIGHTLY	Describes [cat's name] MODERATELY WELL	Describes [cat's name] QUITE WELL	Really DOES describe [cat's name]
Apprehensive	○	○	○	○	○
Clumsy	○	○	○	○	○
Serious	○	○	○	○	○
Faithful	○	○	○	○	○
Cautious	○	○	○	○	○
Gullible	○	○	○	○	○
Nervous	○	○	○	○	○
Independent	○	○	○	○	○
Confused	○	○	○	○	○
Curious	○	○	○	○	○
Loud	○	○	○	○	○
Energetic	○	○	○	○	○
Persistent	○	○	○	○	○
Peaceful	○	○	○	○	○
Timid	○	○	○	○	○
Needy	○	○	○	○	○
Cooperative	○	○	○	○	○
Quick	○	○	○	○	○

Proud	○	○	○	○	○
Domineering	○	○	○	○	○
Charming	○	○	○	○	○
Warm	○	○	○	○	○
Mischievous	○	○	○	○	○
Playful	○	○	○	○	○
Foolish	○	○	○	○	○
Persevering	○	○	○	○	○
Confident	○	○	○	○	○
Territorial	○	○	○	○	○
Demanding	○	○	○	○	○

How hard is it to look after [cat's name]?

- ○ Very easy
- ○ Easy
- ○ Neither hard nor easy
- ○ Hard
- ○ Very hard

To what extent do you agree or disagree with the following statements.

	Strongly disagree	Disagree	Neither agree nor disagree	Agree	Strongly Agree
[cat’s name] gives me a reason to get up in the morning.	◯	◯	◯	◯	◯
There are major aspects of owning a cat that I don't like.	◯	◯	◯	◯	◯
I wish [cat’s name] and I never had to be apart.	◯	◯	◯	◯	◯
[cat’s name] makes too much mess.	◯	◯	◯	◯	◯
It bothers me that [cat’s name] stops me from doing things I enjoyed before I owned him/her.	◯	◯	◯	◯	◯
It is annoying that sometimes I have to change my plans because of [cat’s name].	◯	◯	◯	◯	◯
[cat’s name] costs me too much money.	◯	◯	◯	◯	◯
I would like to have [cat’s name] near me all the time.	◯	◯	◯	◯	◯
If everyone else left me, [cat’s name] would still be there for me.	◯	◯	◯	◯	◯
[cat’s name] helps me get through tough times.	◯	◯	◯	◯	◯

[cat's name] provides me with constant companionship.	○	○	○	○	○
[cat's name] is there whenever I need to be comforted.	○	○	○	○	○

How often do you...?

	Never	Once a month	Once a week	Once every few days	At least once a day
Kiss [cat's name]	○	○	○	○	○
Play games with [cat's name]	○	○	○	○	○
Talk to [cat's name]	○	○	○	○	○
Cuddle [cat's name]	○	○	○	○	○
Have [cat's name] with you while relaxing, i.e. watching TV	○	○	○	○	○
Pet [cat's name]	○	○	○	○	○

How often do you...?

	Never	Once a year	Once a month	Once a week	Once a day
Tell [cat’s name] things you wouldn't tell anyone else	○	○	○	○	○
Feel like looking after [cat’s name] is a chore	○	○	○	○	○
Feel like [cat’s name] stops you from doing things you want to	○	○	○	○	○
Feel like having a cat is more trouble than it's worth	○	○	○	○	○

How often do you spend time enjoying watching [cat’s name]?

- ○ Never
- ○ A couple of times a year
- ○ Once a month
- ○ Once a week
- ○ At least once a day

How often do you buy [cat’s name] presents?

- ○ Never
- ○ A couple of times a year
- ○ Once a month
- ○ Once a fortnight
- ○ Once a week

How traumatic do you think it will be for you when [cat’s name] dies?

- Very untraumatic
- Untraumatic
- Neither traumatic or untraumatic
- Traumatic
- Very traumatic

Do you intend on getting a cat in the next 6 months?

- Yes
- No

How likely would it be that you would keep your **next** cat exclusively indoors from when you acquired it?

- Extremely unlikely
- Unlikely
- Neither likely nor unlikely
- Likely
- Extremely likely

To what extent do you agree or disagree with the following statements about keeping your **next cat** indoors at all times?

	Strongly disagree	Disagree	Neither agree nor disagree	Agree	Strongly agree
I am confident that I could provide enrichment to ensure my next cat was happy indoors	○	○	○	○	○
I am confident I could contain my next cat indoors or to a cat run at all times	○	○	○	○	○

What would encourage you to keep your **next** cat indoors at all times? *(Please write)*

____

Have you ever lost a cat to: *(please tick all that apply)*

- ☐ Car accident
- ☐ Feline Immunodeficiency Virus (FIV)
- ☐ Dog attack
- ☐ Human attack
- ☐ Skin cancer
- ☐ Injury from wildlife (e.g. snakebite)
- ☐ Ingesting poisons (e.g. rat bait)
- ☐ Falling from a height (e.g. out of a window or tree, or off a ledge)
- ☐ Unknown, my cat never came home
- ☐ Other (please write) ____
- ☐ None of the above

If you intend to allow your **next** cat to roam outdoors, which of the following could make you want to exclusively keep him/her indoors?

	Extremely unlikely	Unlikely	Neither likely nor unlikely	Likely	Extremely likely
Improving your cat's mental health	○	○	○	○	○
Improving your cat's physical health	○	○	○	○	○
Increasing your cat's life span	○	○	○	○	○
Helping native wildlife	○	○	○	○	○
Reducing risk of harm to your cat from other humans or dogs	○	○	○	○	○
Reducing risk of harm to your cat from wildlife	○	○	○	○	○
Giving you more one-on-one time with your cat	○	○	○	○	○
Improving your cat's health	○	○	○	○	○

To what extent do you agree or disagree with the following statements.
Cats in general are...?

	Strongly disagree	Disagree	Neither agree nor disagree	Agree	Strongly agree
Loyal	○	○	○	○	○
Affectionate	○	○	○	○	○
Intelligent	○	○	○	○	○
Lovable	○	○	○	○	○
Good company	○	○	○	○	○
Aggressive	○	○	○	○	○
Playful	○	○	○	○	○
Spiteful	○	○	○	○	○
Friendly	○	○	○	○	○
Useful	○	○	○	○	○
Aloof	○	○	○	○	○
Cuddly	○	○	○	○	○
Independent	○	○	○	○	○
Low maintenance	○	○	○	○	○
Expensive	○	○	○	○	○
Spiritual	○	○	○	○	○
Family members	○	○	○	○	○
Dirty	○	○	○	○	○
Noisy	○	○	○	○	○

Fun	○	○	○	○	○
Naughty	○	○	○	○	○

Approximately what proportion of your family and friends own a cat?

- ○ None
- ○ Less than half
- ○ About half
- ○ More than half
- ○ All

If any of your family and friends own cats, which best describes how most of them are kept?

- ○ Indoors only
- ○ Outdoors only
- ○ Indoors at night, outdoors during the day
- ○ Have unrestricted access to the indoors and outdoors

To what extent do you agree or disagree with the following statements about ***cats in general?***

	Strongly disagree	Disagree	Neither agree nor disagree	Agree	Strongly agree
Hunting is a natural part of a cat's behaviour	○	○	○	○	○
It is important for a cat's wellbeing that it is allowed to hunt	○	○	○	○	○
If a cat is well cared for there is no need to allow it outside to hunt	○	○	○	○	○

To what extent do you agree or disagree with the following statements that all pet cats:

	Strongly disagree	Disagree	Neither agree nor disagree	Agree	Strongly agree
Should be kept indoors or in a cat run at **night time**	○	○	○	○	○
Should be kept indoors or in a cat run **at all times**	○	○	○	○	○

To what extent do you agree or disagree with the following statements. The keeping of pet cats **indoors or in a cat run** at all times...

	Strongly disagree	Disagree	Neither agree nor disagree	Agree	Strongly agree
Keeps them safe	○	○	○	○	○
Is unnatural	○	○	○	○	○
Is important to protect wildlife	○	○	○	○	○
Is difficult	○	○	○	○	○
Should be required by law	○	○	○	○	○
Prevents them from being a nuisance to others	○	○	○	○	○
Is not important	○	○	○	○	○
Is expensive	○	○	○	○	○
Is good for the cat's health and wellbeing	○	○	○	○	○
Is the right thing to do	○	○	○	○	○
Is a practice that members of my household would agree with	○	○	○	○	○
Is a practice my neighbours would agree with	○	○	○	○	○
Is a practice my family and friends would agree with	○	○	○	○	○

Is a practice that my veterinarian would agree with	○	○	○	○	○

To what extent do you agree or disagree with the following statement that all adult pet cats:

	Strongly disagree	Disagree	Neither agree nor disagree	Agree	Strongly agree
Should be registered with a local council	○	○	○	○	○

To what extent do you agree or disagree with the following statement that the registration of adult pet cats:

	Strongly disagree	Disagree	Neither agree nor disagree	Agree	Strongly agree
Keeps them safe	○	○	○	○	○
Helps fund important animal services	○	○	○	○	○
Makes it easier to find their owner if they become lost	○	○	○	○	○
Is difficult	○	○	○	○	○
Should be required by law	○	○	○	○	○
Is unnecessary	○	○	○	○	○
Is important	○	○	○	○	○
Is expensive	○	○	○	○	○
Is the right thing to do	○	○	○	○	○
Is a practice that members of my household would agree with	○	○	○	○	○
Is a practice that my neighbours would agree with	○	○	○	○	○
Is a practice that my family and friends would agree with	○	○	○	○	○
Is a practice that my veterinarian would agree with	○	○	○	○	○

To what extent do you agree or disagree with the following statement that all pet cats:

	Strongly disagree	Disagree	Neither agree nor disagree	Agree	Strongly agree
Should be microchipped	○	○	○	○	○

To what extent do you agree or disagree that the microchipping of pet cats:

	Strongly disagree	Disagree	Neither agree nor disagree	Agree	Strongly agree
Keeps them safe	○	○	○	○	○
Is unnatural	○	○	○	○	○
Makes it easier to find their owner if they become lost	○	○	○	○	○
Is difficult	○	○	○	○	○
Should be required by law	○	○	○	○	○
Is unnecessary	○	○	○	○	○
Is important	○	○	○	○	○
Is expensive	○	○	○	○	○
Is the right thing to do	○	○	○	○	○
Is a practice that members of my household would agree with	○	○	○	○	○
Is a practice that my neighbours would agree with	○	○	○	○	○
Is a practice that my family and friends would agree with	○	○	○	○	○
Is a practice that my veterinarian would agree with	○	○	○	○	○

To what extent do you agree or disagree with the following statement that all pet cats:

	Strongly disagree	Disagree	Neither agree nor disagree	Agree	Strongly agree
Should be up to date with their vaccinations	○	○	○	○	○

To what extent do you agree or disagree that vaccinating pet cats:

	Strongly disagree	Disagree	Neither agree nor disagree	Agree	Strongly agree
Is good for their health and wellbeing	○	○	○	○	○
Is unnatural	○	○	○	○	○
Is only worthwhile if cats are in contact with other cats	○	○	○	○	○
Is difficult	○	○	○	○	○
Should be required by law	○	○	○	○	○
Is unnecessary	○	○	○	○	○
Is important	○	○	○	○	○
Is expensive	○	○	○	○	○
Is the right thing to do	○	○	○	○	○
Is a practice that members of my household would agree with	○	○	○	○	○
Is a practice that my family and friends would agree with	○	○	○	○	○
Is a practice that my veterinarian would agree with	○	○	○	○	○

To what extent do you agree or disagree with the following statement that all pet cats:

	Strongly disagree	Disagree	Neither agree nor disagree	Agree	Strongly agree
Should be desexed/neutered/spayed	○	○	○	○	○

To what extent do you agree or disagree that desexing/neutering/spaying pet cats:

	Strongly disagree	Disagree	Neither agree nor disagree	Agree	Strongly agree
Helps reduce behavioural problems	○	○	○	○	○
Is only worthwhile if cats are in contact with other cats	○	○	○	○	○
Is unnatural	○	○	○	○	○
Is good for their health	○	○	○	○	○
Is difficult	○	○	○	○	○
Should be required by law	○	○	○	○	○
Is unnecessary	○	○	○	○	○
Is important	○	○	○	○	○
Is expensive	○	○	○	○	○
Prevents them from being a nuisance to others	○	○	○	○	○
Is the right thing to do	○	○	○	○	○
Is a practice that members of my household would agree with	○	○	○	○	○
Is a practice my neighbours would agree with	○	○	○	○	○

Is a practice that my family and friends would agree with	○	○	○	○	○
Is a practice my veterinarian would agree with	○	○	○	○	○

To what extent do you agree or disagree with the following statement that all pet cats:

	Strongly disagree	Disagree	Neither agree nor disagree	Agree	Strongly agree
Should be allowed to express natural behaviours	○	○	○	○	○

To what extent do you agree or disagree that allowing pet cats to express natural behaviours:

	Strongly disagree	Disagree	Neither agree nor disagree	Agree	Strongly agree
Helps reduce behavioural problems	○	○	○	○	○
Is unnatural	○	○	○	○	○
Is good for their health	○	○	○	○	○
Is difficult	○	○	○	○	○
Is unnecessary	○	○	○	○	○
Is important	○	○	○	○	○
Is the right thing to do	○	○	○	○	○
Is a practice that members of my household would agree with	○	○	○	○	○
Is a practice that my family and friends would agree with	○	○	○	○	○
Is a practice that my veterinarian would agree with	○	○	○	○	○

To what extent do you agree or disagree with the following statement that all pet cats:

	Strongly disagree	Disagree	Neither agree nor disagree	Agree	Strongly agree
Should be fed a nutritionally balanced diet	○	○	○	○	○

To what extent do you agree or disagree that feeding pet cats a nutritionally balanced diet:

	Strongly disagree	Disagree	Neither agree nor disagree	Agree	Strongly Agree
Is good for their health	○	○	○	○	○
Is unnatural	○	○	○	○	○
Maintains ideal body weight and condition	○	○	○	○	○
Is difficult	○	○	○	○	○
Makes them friendly	○	○	○	○	○
Prevents them from being a nuisance to others	○	○	○	○	○
Is necessary	○	○	○	○	○
Is not important	○	○	○	○	○
Is expensive	○	○	○	○	○
Prevents them from hunting	○	○	○	○	○
Is the right thing to do	○	○	○	○	○
Is a practice that members of my household would agree with	○	○	○	○	○
Is a practice my friends and family would agree with	○	○	○	○	○
Is a practice that my veterinarian would agree with	○	○	○	○	○

To what extent do you agree or disagree with the following statement that all pet cats:

	Strongly disagree	Disagree	Neither agree nor disagree	Agree	Strongly agree
Should visit the vet annually for a health checkup	○	○	○	○	○

To what extent do you agree or disagree that taking pet cats to the vet for annual health check ups:

	Strongly disagree	Disagree	Neither agree nor disagree	Agree	Strongly agree
Is good for their health	○	○	○	○	○
Is unnatural	○	○	○	○	○
Is difficult	○	○	○	○	○
Is necessary	○	○	○	○	○
Is not important	○	○	○	○	○
Is expensive	○	○	○	○	○
Is the right thing to do	○	○	○	○	○
Is a practice that members of my household would agree with	○	○	○	○	○
Is a practice that my friends and family would agree with	○	○	○	○	○
Is a practice that my veterinarian would agree with	○	○	○	○	○

From which of the following sources would/do you get information about appropriate cat keeping and care? *(Please select all that apply)*

- ☐ Veterinarian
- ☐ RSPCA
- ☐ Pet Shop
- ☐ Friends
- ☐ Family
- ☐ Zoos Victoria
- ☐ General Google Search
- ☐ Social media
- ☐ Local Council
- ☐ Other cat shelter or rescue (e.g. Cat Protection Society, Lost Dogs Home)
- ☐ Cat Breeder
- ☐ Cat Fancier Association
- ☐ Books
- ☐ Other (specify) ____

How likely would it be that you would act on advice given to you about a/your cat from the following?

	Extremely unlikely	Unlikely	Neither likely nor unlikely	Likely	Extremely likely
Veterinarian	○	○	○	○	○
RSPCA	○	○	○	○	○
Pet Shop	○	○	○	○	○
Friends	○	○	○	○	○
Family	○	○	○	○	○
Zoos Victoria	○	○	○	○	○
General Google search	○	○	○	○	○
Social Media	○	○	○	○	○
Local Council	○	○	○	○	○
Other cat shelter or rescue (e.g. Cat Protection Society, Lost Dogs Home)	○	○	○	○	○
Cat Breeder	○	○	○	○	○
Cat Fancier Association	○	○	○	○	○
Books	○	○	○	○	○
Other (specify)	○	○	○	○	○

How likely is it that you will do the following with [cat’s name] in the future?

	Extremely unlikely	Unlikely	Neither likely nor unlikely	Likely	Extremely likely
I intend to keep [cat’s name] indoors or in a cat run all the time this month	○	○	○	○	○
I intend to start keeping [cat’s name] indoors or in a cat run more frequently this month	○	○	○	○	○

Are you:

- ○ Male
- ○ Female
- ○ Non-binary/third gender
- ○ Prefer not to say

What is your postcode?

____

In what year were you born?

____

What is your *household* annual income from all sources, before taxes?

- ◯ Less than $30,000
- ◯ $30,001 to $50,000
- ◯ $50,001 to $70,000
- ◯ $70,001 to $90,000
- ◯ $90,001 to $120,000
- ◯ $120,001 to $150,000
- ◯ $150,001 to $200,000
- ◯ $200,001 plus
- ◯ Prefer not to say

Which of the following best describes your employment status?

- ◯ Retired
- ◯ Unemployed
- ◯ Unable to work
- ◯ Engaged in home duties
- ◯ Casual paid work
- ◯ Part time paid work (30 hours or less per week or seasonal work)
- ◯ Full time paid work (31+ hours a week)
- ◯ Student
- ◯ Other (write) ____
- ◯ Prefer not to say

Which of the following best describes your current occupation?

- ○ None
- ○ Farm owner or manager
- ○ Manager (Sales manager)
- ○ Researcher
- ○ Professional (e.g. Dietician, Accountant, Teacher)
- ○ Technician or Trade worker (e.g. plumber)
- ○ Community or Personal Service (e.g. child carer)
- ○ Clerical or administration work (e.g. receptionist, data entry)
- ○ Sales (e.g. sales assistant, sales representative)
- ○ Machine operator (e.g. truck driver, storeperson)
- ○ Labourer (e.g. cleaner, gardener, farm labourer)
- ○ Other ____
- ○ Prefer not to say

What is your *highest* level of education?

- ◯ Primary School
- ◯ Secondary School
- ◯ TAFE College
- ◯ University (Undergraduate degree)
- ◯ University (Postgraduate degree)
- ◯ No formal schooling
- ◯ Other ____
- ◯ Prefer not to say

Which of the following best describes where you live?

- ◯ Inner city
- ◯ Inner suburban
- ◯ Outer suburban
- ◯ Major regional city
- ◯ Small regional town
- ◯ Semi-rural
- ◯ Rural
- ◯ Remote

In what kind of dwelling do you currently live?

- ○ House with large outside space (4 acres and up)
- ○ House with medium outside space (large garden or less than 4 acres)
- ○ House with small outside space (patio or small garden/courtyard)
- ○ Semi-detached, terrace or townhouse with medium outside space (large garden)
- ○ Semi-detached, terrace or townhouse with small outside space (patio or small garden/courtyard)
- ○ Flat, unit, apartment with small outside space (balcony or patio)
- ○ Flat, unit, apartment with no outside space
- ○ Other ____

Which of the following best describes your ownership of this dwelling?

- ○ I or my family owns or is paying it off
- ○ I or my family rent it
- ○ Other ____

Which of the following people usually live with you? (*Please tick all that apply)*

- ☐ Partner (spouse or de facto)
- ☐ One or more adults over 65 years
- ☐ One or more adults between 18 and 65 years
- ☐ One or more children between 12-18 years
- ☐ One or more children under 12
- ☐ Other

In the last 12 months how many times have you visited Melbourne Zoo, Werribee Open Range Zoo, or Healesville Sanctuary?

- ○ Never
- ○ Once
- ○ A few times
- ○ More than 5 times

When were you last at the Zoo?

____

Do you have a Zoos Victoria membership?

- ○ Yes
- ○ No
